# Computed tomographic arthrography, gross anatomy and histology demonstrate a communication between synovial invaginations in the proximal aspect of the third interosseous muscle and the carpometacarpal joint in horses

**DOI:** 10.3389/fvets.2022.958598

**Published:** 2022-09-02

**Authors:** Carolin Gerdes, Rhiannon Morgan, Rebecca Terry, Alastair Foote, Roger Smith

**Affiliations:** ^1^Pferdeklink Hochmoor GmbH, Gescher, Germany; ^2^The Royal Veterinary College, London, United Kingdom; ^3^Rossdales Laboratories, Newmarket, United Kingdom

**Keywords:** proximal suspensory ligament, carpus, computed tomography, arthrography, synovial communication

## Abstract

This descriptive anatomical study investigates the relationship between the third interosseous muscle, also known as the suspensory ligament, and the carpometacarpal joint in forelimbs of horses, with the hypothesis that there was a direct synovial communication between these structures as shown by computed tomographic arthrography, histology, and gross anatomy sections. Computed tomography of the carpus and metacarpal region was performed on two groups. Group 1 consisted of eight cadaver limbs undergoing computed tomographic arthrography following injection of a mixture of positive contrast medium, saline, and color-pigmented fluid solution into the middle carpal joint. Group 2 consisted of eight forelimbs assessed using plain computed tomography. The images were interpreted subjectively for contrast medium distribution and objectively by comparing Hounsfield values of the proximal suspensory ligament at 0.5 cm intervals starting at the origin and extending 3 cm distal to the proximal subchondral bone plate of the third metacarpal bone. Of the 16 limbs, two were sectioned for gross anatomy and one was documented histologically. The proximal suspensory ligament was visualized with clear margins on computed tomography images. The positive contrast medium was found within the lateral and medial lobes of the suspensory ligament in all eight (100%) limbs. Hounsfield units within the suspensory ligament following contrast injection were significantly higher than in those in the plain CT group between 0.5 and 2.5 cm distal to the proximal subchondral bone plate of the third metacarpal bone (*p* < 0.05). The gross anatomy sections showed color pigmentation within the suspensory ligament correlating to the contrast medium distribution evident on computed tomography images. Histology confirmed a synovial lined cavity within the suspensory ligament. The demonstration of a direct synovial communication between the internal structure of the proximal suspensory ligament and the carpometacarpal joint in horses offers further explanation for commonly encountered interactions of diagnostic local anesthesia of the carpal and subcarpal regions. When performing diagnostic or therapeutic injections into the middle carpal joint, the likely effect on the proximal suspensory ligament should be considered. Furthermore, as the proximal suspensory ligament was identified clearly on CT images, further studies are needed to elucidate the utility of CT in clinical cases with suspected soft tissue pathology in the subcarpal region.

## Introduction

Lameness and poor performance originating from the carpus and/or the proximal aspect of the third interosseous muscle (also named the proximal aspect of the suspensory ligament, PSL) in the forelimb is a common problem in equine athletes (1, 2). Due to the complex architecture of the PSL, the deep position and its close proximity to distal outpouchings of the carpal joints, diagnosis can be challenging.

The description of the forelimb PSL anatomy in the literature, with regard to its origin, varies in some of its details. There is a consensus that the main part of the suspensory ligament originates from the proximopalmar aspect of the third metacarpal bone (Mc3). Additional fibers attach further proximal to the fourth and third carpal bones (3, 4), to the palmar carpal ligament (5, 6), and medially and laterally to the proximoaxial surfaces of the second and fourth metacarpal bones (7). The PSL consists of collagenous (fibrous), adipose, and muscle tissues and has a large variability in its structure in normal horses (3, 4).

The middle carpal joint (MCJ) always communicates with the carpometacarpal joint (8), which has lateral and medial distopalmar outpouchings located close to the axial aspect of the second and fourth metacarpal bones (9). Inadvertent anesthesia of the carpometacarpal joint (and therefore the middle carpal joint) when performing high palmar perineural nerve blocks, and vice versa, is common and has previously been explained by the injection of these outpouchings (1, 10, 11). Gray et al. (12) have found computed tomographic arthrography useful to visualize intercarpal ligaments and also demonstrated synovial outpouchings into the PSL following injection of several synovial cavities concurrently, including the carpal sheath and both the antebrachiocarpal and middle carpal joints. However, to the authors' knowledge, no study has investigated the synovial outpouchings of the carpometacarpal joint within the PSL by comparing computed tomographic arthrography of the middle carpal joint with plain CT images, anatomic sections, and histology.

Therefore, the objective of this study was to describe the relationship between the PSL and the carpometacarpal joint by comparing plain computed tomography (CT) and CT arthrography following injection of the middle carpal joint. On a small number of limbs, histology (*n* = 1) and gross anatomy sections (*n* = 2) were used to confirm synovial outpouchings within the PSL. It was hypothesized that 1) positive contrast medium will be found within the proximal suspensory ligament, suggesting a direct synovial communication between the two structures, and 2) the synovial invagination could be identified histologically and on gross anatomy sections within the suspensory ligament correlating to the outpouching of the carpometacarpal joint.

## Materials and methods

### Animals

A total of 16 equine forelimbs from different cohorts were examined in two groups. The horses used for this study were from a clinical hospital population.

#### Group 1

In total, eight forelimbs were transected at the elbow joint immediately after euthanasia from seven horses for reasons unrelated to lameness affecting the examined sites. Written informed consent was given by the owners with the possibility to opt out of postmortem research. Imaging was performed within 2 hours of euthanasia.

#### Group 2

A total of eight forelimbs of five horses from a different cohort were used as a reference to be compared to group 1 and acted as controls. The horses previously underwent a lameness workup including diagnostic local anesthesia and standard imaging (radiography and/or ultrasound) without any signs or a known history of pathology or lameness associated with the proximal suspensory ligament or carpal region. The horses underwent plain computed tomography of the forelimb as part of an orthopedic examination.

### Computed tomography

#### Computed tomographic arthrography of equine cadaver limbs (*ex vivo* group 1)

Computed tomographic arthrography of the carpometacarpal and middle carpal joints was performed on eight cadaver limbs (group 1). A measure of 10 ml of a mixture containing 5 ml of positive radiographic contrast medium, 300 mg iodine per milliliter non-ionic monomer iohexol (Omnipaque ^A^ GE Healthcare AS, Oslo, Norway), 3 ml of 0.9% NaCl saline solution, and 2 ml of methylene blue were injected into the middle carpal joint. The injection was administered shortly after euthanasia *via* the dorsal approach using a 20-gauge, 1$\frac{1}{2}$-inch needle in a partially flexed leg position after synovial fluid was aspirated to confirm correct needle placement into the joint. Following the injection, the limbs were flexed and extended 10 times to ensure maximum possible distribution of the medium. The limbs were positioned in longitudinal orientation and parallel to the CT table. Post-contrast images were acquired using a 16-slice multi-detector CT scanner (GE Medical, Lightspeed16, Chicago, Illinois, United States) with 0.625 mm slice thickness, 512x512 pixel matrix, 120 kV, and 160 mA. The images were processed using a standard bone algorithm.

#### Plain computed tomography of equine limbs (*in vivo* group 2)

Plain computed tomography images of eight forelimbs of five horses imaged under general anesthesia were reviewed (group 2). Each horse was premedicated with romifidine (0.08 mg/kg body weight, intravenously), and general anesthesia was induced by administering a combination of ketamine (2 mg/kg body weight, intravenously) and diazepam (0.05 mg/kg body weight, intravenously). Intravenous anesthesia was maintained using a romifidine-based triple drip combination of romifidine (0.05 mg/ml) and ketamine (2 mg/ml) in 500 ml 10% guaifenesin. The horses were positioned in lateral recumbency on a custom-built CT table with one or both forelimbs positioned along the axis of the CT table and perpendicular to the gantry using positioning aids such as velcro fastener straps and foam pads. Images were acquired using a 16-slice multi-detector CT scanner (Siemens Somatom Sensation, Siemens Medical Solutions, Erlangen, Germany). Continuous transverse images of 0.75 mm slice thickness, 512×512 pixel matrix, 140 kV, and 260 mA were obtained from the carpus to the distal phalanx and processed using standard bone and soft tissue algorithms.

### CT image analysis

CT arthrography and plain CT images from two different cohorts were evaluated using a DICOM viewer (Horos, Bernex, Switzerland), by one orthopedic clinician (CG) and one European College of Veterinary Diagnostic Imaging board-certified radiologist (REM). The positive contrast medium distribution within the palmar soft tissues of the distal carpus and proximopalmar metacarpus was described by consensus (CG and REM), and abnormal findings affecting any structures in the carpal and subcarpal regions were recorded. Multiplanar reformatting tools were used to inspect the contrast medium distribution within the proximal suspensory ligament in sagittal, transverse, and dorsal planes.

**Figure 1:**
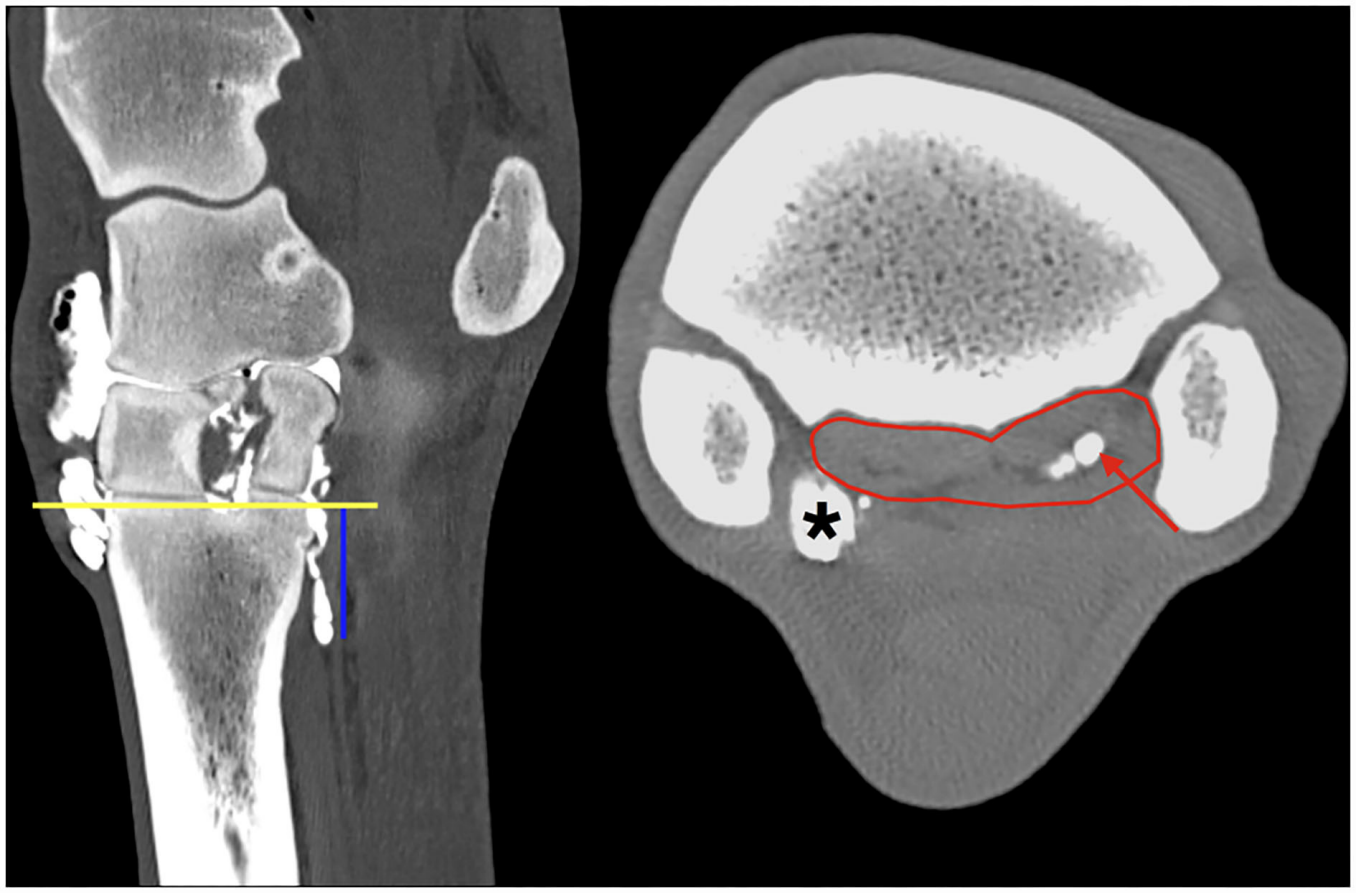
Multiplanar reconstruction (MPR) of the CT arthrogram in transverse (right) and sagittal (left) planes following injection of positive contrast medium into the middle carpal joint; medial and dorsal are to the left. The region of interest is defined by a polygon (red line) outlining the borders of the PSL at the level 2.5 cm (blue vertical line) distal to the proximal subchondral bone plate of the third metacarpal bone (yellow horizontal line). Note the positive contrast medium (red arrow) is evident in the center of the lateral lobe only, in the region of the muscle and fat tissue bundles, at this level of the PSL. Particularly, the medial palmarodistal outpouching of the carpometacarpal joint is evidently filled with positive contrast medium in the periphery (asterisk) adjacent to the axial aspect of the SL.

For the objective assessment, a closed polygon was placed to outline the suspensory ligament on transverse images. The mean Hounsfield units (HU) were recorded for this region by one author (CG). Following a standardized protocol, the measurements were taken over 6 cm at 0.5 cm intervals starting at 0.5 cm distal to the proximal subchondral bone plate of Mc3. The placement of the measurements can be seen in Figure 1. The presence and location of synovial invaginations within the PSL were documented, the proximodistal extent of the contrast medium distribution into the PSL was measured, and a mean value for the distal extent of the contrast medium was calculated. The measurements of the contrast medium within the lateral and medial lobes were compared.

Objective evaluation and measurement of the proximal suspensory ligament was performed on all limbs in both groups (*n* = 16). For each forelimb, 12 measurements were taken, resulting in a total of 192 measurements for both groups.

For the assessment of intra-observer variability, measurements of limbs 1, 3, and 5 (group 1) and controls (group 2) 1, 3, and 5 were repeated five times at level 1 cm, 2 cm, and 3 cm distal to the proximal subchondral bone plate of Mc3 by the same author (CG) 2 weeks apart.

### Gross anatomy specimen and histology

Immediately after image acquisition, two cadaver limbs of group 1 were frozen. A band saw was used to transect the limb into several slices of approximately 0.5 cm thickness concentrating on the region of interest between the carpometacarpal joint and 6 cm distal to that in sagittal planes. For orientation, the previously acquired CT images were used. The slices were photographed. An additional limb out of group 1 was prepared for histology. The region of interest was chosen according to the distribution of methylene blue within the SL and the CT images. The regions of interest were dissected and fixed in 10% neutral buffered formalin, embedded in paraffin, and sectioned. Hematoxylin and eosin (HE)-stained sections were evaluated by two Royal College of Veterinary Surgeons recognized specialists in pathology (AF and RT).

### Statistical analysis

To determine intra-observer variability, the coefficient of variation (CV) in relation to the HU measurement mean of six repeatedly measured transverse images of the PSL was calculated (%CV) (13, 14).

Linear mixed effects model was used to evaluate the effects of positive contrast medium within the MCJ on the measurement of the SL HU at each location; first-degree autoregressive structure was used to account for repeated measures at each level, and random effect of the forelimb was accounted for pre- and post-contrast (*in vivo* plain CT group 2 and *ex vivo* CT arthrography group 1) repeated measurements at each level. Fisher's least significant difference (LSD) was used for *post hoc* comparison.

*T*-test was performed to compare the age between the two cohorts, CT arthrography and plain CT, for similarity.

SPSS statistics (IBM, International Business Machines Corporation, New York, USA) was used as a software tool to compute statistics.

## Results

### Horses

The horses in the *ex vivo* group 1 (*n* = 7) had a mean age of 10.3 (range 5–19, median 8) years. They were of different breeds (five Warmbloods, one Thoroughbred, and one Spanish crossbreed) and sex (four geldings and three mares). The horses in the *in vivo* group 2 (*n* = 5) had a mean age of 8.8 (range 5–11, median 10) years. The breeds were warmbloods (*n* = 3) and Quarter horses (*n* = 2): three geldings and two mares. Comparing the groups regarding the age of the horses, there was no significant difference (*p* = 0.23), showing a similarity between the two cohorts.

### Computed tomography

The outlines of the PSL were well defined on all images using both bone and soft tissue windows. The bilobed architecture and the heterogenous structure with hyperattenuating parts on the periphery and hypoattenuating tissue in the center of each lobe could be identified. The SL could be clearly differentiated from surrounding soft tissues with appropriate window and leveling (approximate values used in this study: level of 70-150 HU and window of 200-600 HU), as well as following the SL using MPR repeatedly from proximal to distal.

Subjective image analysis of all limbs in groups 1 and 2 revealed no obvious irregularities affecting the proximal suspensory ligament or any of the other soft tissue structures in this region. Abnormal findings included enlarged vascular channels in the proximal third metacarpal bone in one limb, sclerosis of the carpal bones (radial facet of the third and dorso-distal aspect of the radial carpal bones) in four limbs, ossification of the interosseous ligament in the region of the second and fourth metacarpal bones in one limb, mild enthesophyte formation on the dorsal aspect of the third carpal bone in one limb, small osteophyte formation at the MCJ in one limb, and a small subchondral cyst-like lesion of the third carpal bone proximally toward the MCJ in one limb.

**Figure 2:**
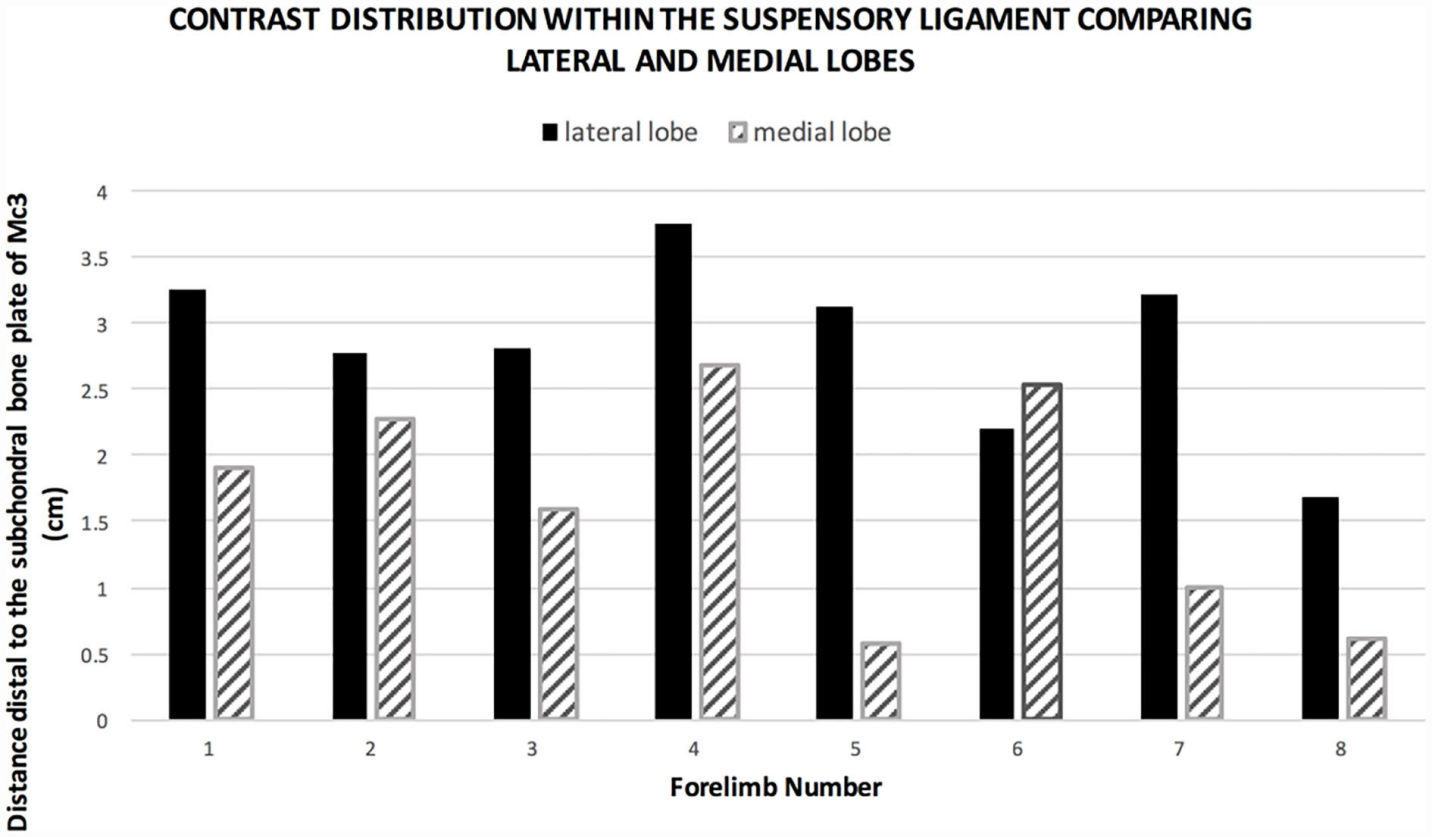
Graph showing the extent of contrast medium distal to the subchondral bone plate in group 1. The positive contrast medium extends further distal in the lateral lobe compared to the medial lobe in seven of eight limbs.

Positive contrast medium was present in all MCJs and CMCJs of the limbs in the *ex vivo* CT arthrography group 1. Distribution of the contrast was evident in both the lateral and the medial lobe of the suspensory ligament in all eight forelimbs (100%). There was a clear distinction between the well-defined contrast pooling and the surrounding tissue (Figure 1). The furthest distal aspect of obvious contrast medium within the SL ended blindly in the center of the lateral lobe in seven of the eight limbs (87.5%), with a mean distance of 2.85 cm (range 1.68–3.76 cm). In only one limb, there was an outpouching ending blindly and furthest distal in the medial lobe. In the medial lobes, the mean distance of the distal extent was 1.65 cm (range 0.62–2.69 cm). Contrast medium in the lateral lobe extended further distally than in the medial lobe in seven of the eight limbs (Figure 2). Contrast medium distribution could be followed from synovial outpouchings entering the SL dorsally extending from either side medially and laterally in between the second, third, and fourth metacarpal bones. The positive contrast medium was localized centrally in the region of the muscle and adipose tissues in each lobe of the SL and had a variable connection to the corresponding palmar carpometacarpal outpouchings. A visible connection to the palmar carpometacarpal joint pouch was present medially in seven of the eight (87.5%) and laterally in six of the eight (75%) limbs. The synovial outpouchings were located centrally in the PSL invading a space within the layer originating from the Mc3, dorsal to the layer originating from the palmar carpal ligament.

There was very low intra-observer variability (1.97% CV) between 30 repeated HU measurements of the SL.

**Figure 3:**
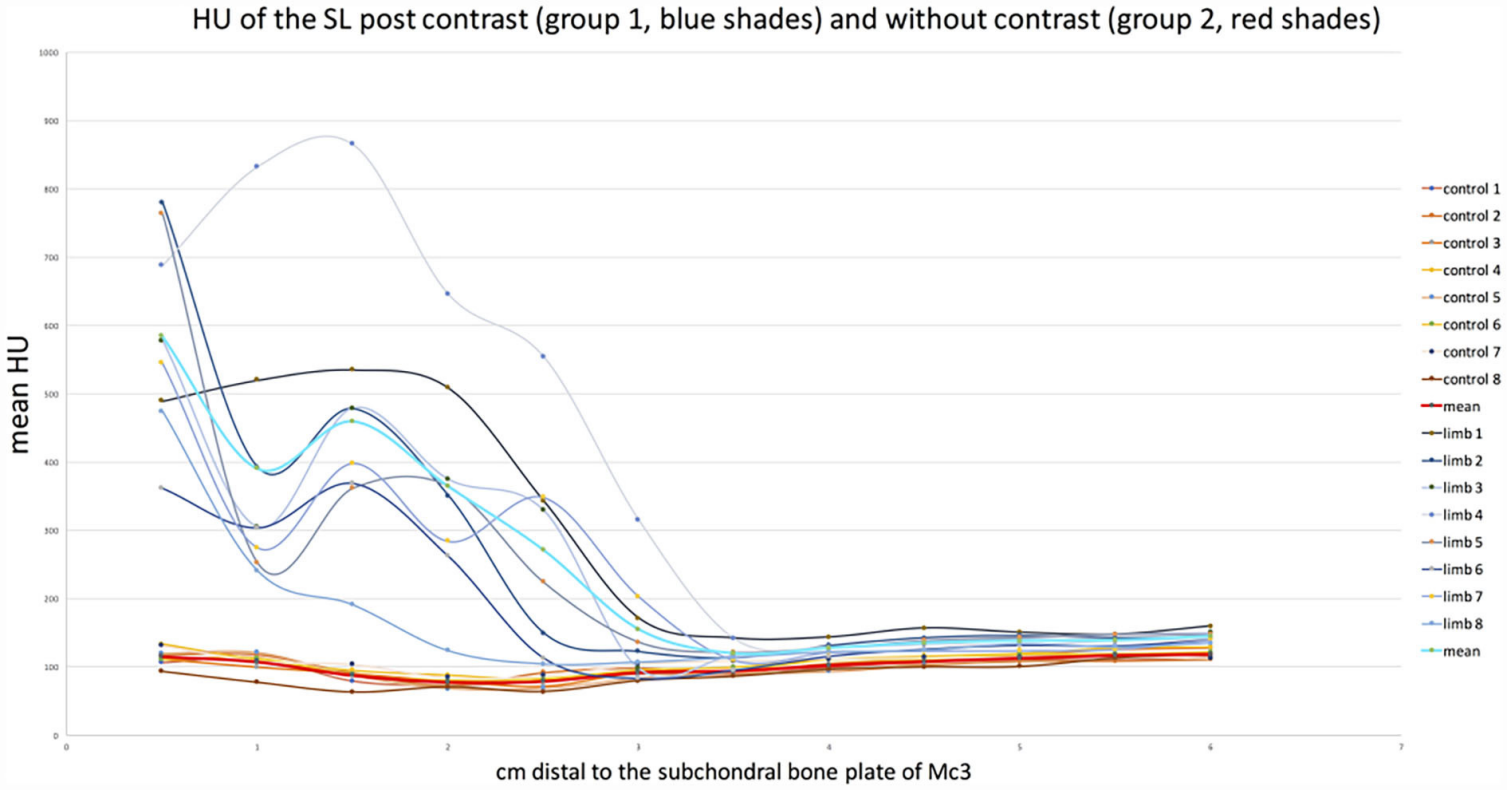
Graph showing the change in mean HU values at each level of both groups. Graphs in blues shades (group 1, with contrast) show the significantly higher values in the proximal region (0.5–2.5 cm) than distally (3–6 cm). The graphs in red shades (group 2, control) show a flat course with similar values on both sides of the dotted line.

CT arthrography HU measurements were significantly higher than reference plain CT HU measurements within the SL between 0.5 and 2.5 cm distal to the proximal subchondral bone plate of the third metacarpal bone (*p* < 0.05). There was no significant difference in HU mean values between the two groups between 3 and 6 cm distal to the subchondral bone plate of the third metacarpal bone (Table 1 and Figure 3). The mean of the HU values was 413.7 HU (±30.81) for the proximal part of the suspensory ligament (0.5–2.5 cm distal to the subchondral bone plate of Mc3) and 136.34 HU (±4.08) for the distal part in the CT arthrography group 1. In the plain CT group 2, the mean of the HU values was 92.72 HU (±3.03) proximally and 106.17 HU (±1.61) distally.

**Table 1:** ROI along the outline of the suspensory ligament in transverse CT images measured in mean HU at 6 levels, from 0.5 cm to 6 cm distal to the subchondral bone plate of Mc3, in 0.5 cm intervals.

**Location (cm)**	**Mean contrast group 1 (HU)**	**Mean plain CT group 2 (HU)**	**Mean difference between groups 1+2 (HU)**
0.5	584.76	115.00	469.765*
1	389.91	107.05	282.856*
1.5	459.30	86.84	372.457*
2	364.06	76.73	287.326*
2.5	270.46	77.96	192.495*
3	154.09	91.39	62.702
3.5	118.98	93.77	25.217
4	126.95	101.92	25.031
4.5	134.40	107.85	26.549
5	137.45	112.66	24.795
5.5	138.34	116.91	21.431
6	144.17	118.68	25.486

^*^ p < 0.05.

### Gross anatomy specimen and histology

**Figure 4:**
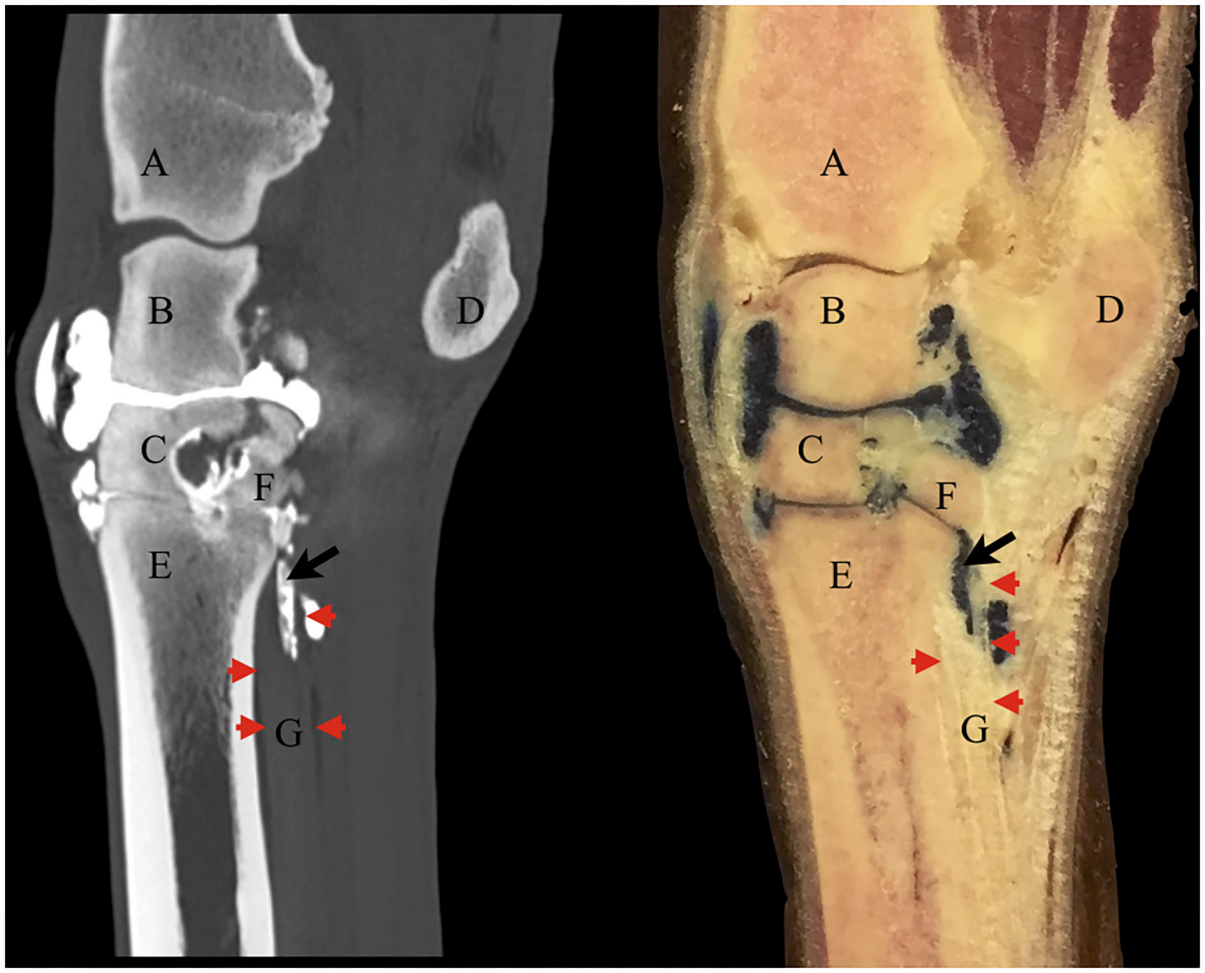
Corresponding contrast CT arthrogram (left) and gross anatomy sagittal section specimen (right) following contrast/methylene blue mixture injection into the middle carpal joint of the same cadaver limb. The red arrowheads point out the dorsal (left) and palmar (right) borders of the origin of the suspensory ligament on both images. There is positive contrast medium on the CT image in the same location as the methylene blue mixture in the anatomical section within the ligament (black arrow). Some of the solution had leaked under the skin dorsal to the middle carpal joint at the injection site. **(A)** distal radius, **(B)** intermediate carpal bone, **(C)** third carpal bone, **(D)** accessory carpal bone, **(E)** third metacarpal bone, **(F)** fourth carpal bone, **(G)** suspensory ligament.

**Figure 5:**
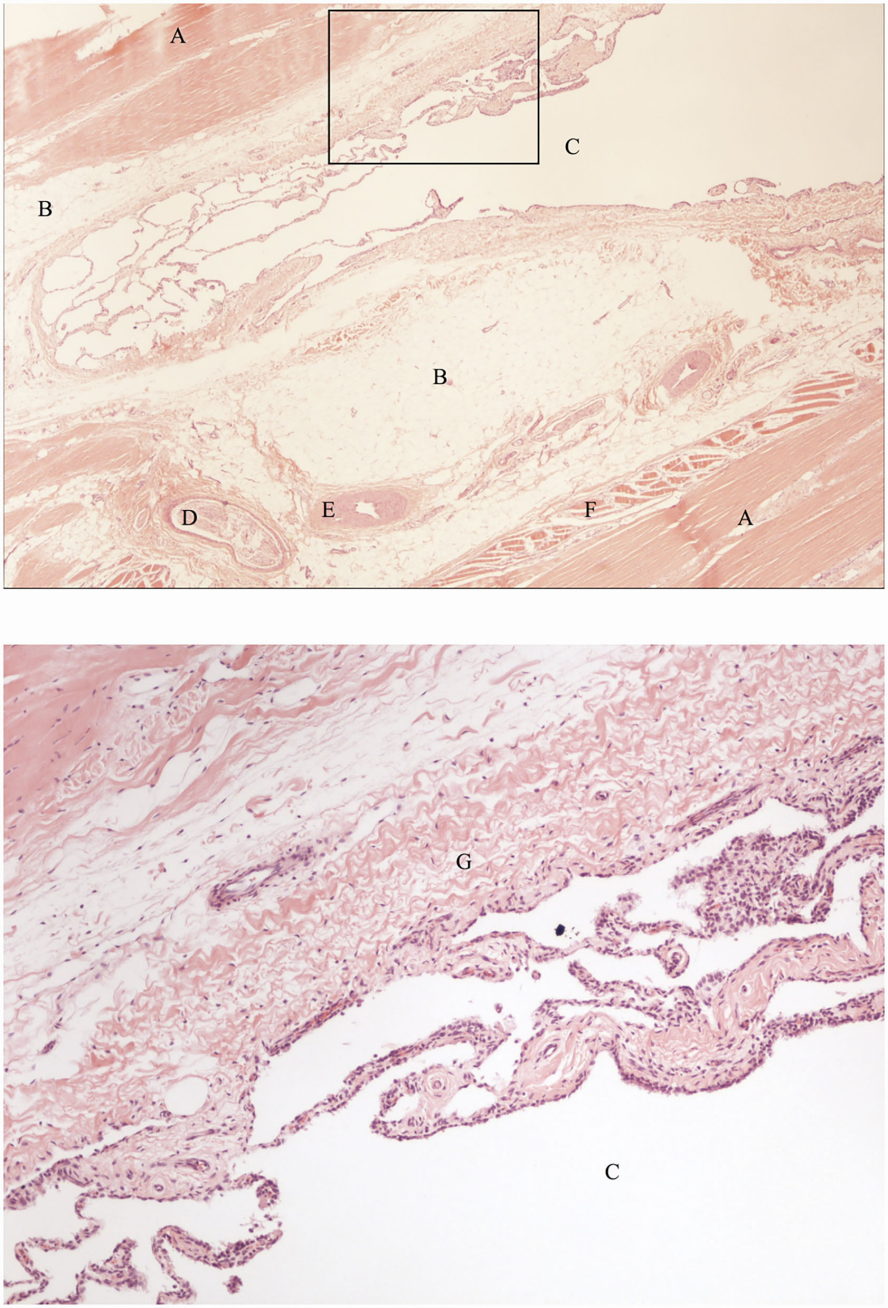
Histological sections of the SL region indicated by the black arrow in Figure 4, showing a synovial lined cavity within the SL (hematoxylin and eosin). The bottom image is an enlarged section of the the image on top (indicated by the black box) showing details of the histological appearance of the synovial lined cavity. A: fibrous tissue, B: adipose tissue, C: synovial lined cavity, D: nerve, E: artery, F: muscle tissue, G: subsynovial connective tissue (subintima).

The contrast/methylene blue mixture was distributed throughout the synovial pouches of the carpometacarpal and middle carpal joints. Within the SL, methylene blue solution was visible extending from the carpometacarpal joint distally and ending blindly, confirming the findings on the sagittal CT images (Figure 4). Histologically, a synovial lined cavity was identified within the SL (Figure 5).

## Discussion

The results of the study prove there is a communication between the carpometacarpal joint and the PSL *via* synovial invaginations not only closely associated with the borders of the PSL but also within the center of the SL lobes in all the limbs assessed. Positive contrast medium was visible within the lateral and medial lobes of the SL on CT images on subjective inspection in all eight horses, which was supported by objective measurement of Hounsfield units showing significantly higher values for the PSL in contrast CT arthrography group 1 compared to plain CT group 2 between the levels 0.5 and 2.5 cm distal to the subchondral bone plate of the carpometacarpal joint. This could have represented diffusion into the ligament substance, but the presence of methylene blue within the PSL in the gross anatomy sections and the evidence of a synovial invagination within the SL in the same location of contrast medium evident on CT images indicate that there is a true synovial extension from the joint within the center of the PSL. This anatomical feature is highly relevant to the interpretation of diagnostic local anesthesia and diagnostic imaging. The results may implicate alternative treatment routes for PSL disease. Before implementing carpal injections as a therapeutic strategy for proximal suspensory injuries, further information regarding the possibilities of achieving distension or filling of the synovial invaginations following injection and possible flow back during flexion of the limb into the main carpal joint is required. Further investigations into injecting the middle carpal joint of horses *in vivo*, ideally in a clinical setting, would allow for determining whether the synovial invaginations are also present or possibly enlarged following the injection of fluid. *In vivo*, the invaginations could be different in size or shape due to muscle fiber contractions in the subintima or due to tension in the ligament during weight bearing.

The depth of the synovial invagination in the lateral lobe had a mean distance of 2.85 cm (range 1.68–3.76 cm) and 1.65 cm (range 0.62–2.69 cm) in the medial lobe. The effect of age of horses was not taken into account in the statistical analysis in this study but could potentially influence the number and depth of the synovial invaginations.

Other authors have found variable evidence of these synovial invaginations previously. Gray et al. (12) demonstrated positive contrast medium in the internal structure of the proximal suspensory ligament following the simultaneous injection of multiple adjacent synovial structures to investigate visualization and characterization of the intercarpal ligaments. Due to injection into the antebrachiocarpal joint, middle carpal joint, and carpal sheath prior to scanning, the exact site of communication could not be elucidated. Ford et al. (9) investigated communications and boundaries of the MCJ and CMCJ by injecting a latex solution into 50 cadaver limbs. They found small outpouchings extending into the fibers of the suspensory ligament axially. Pooling of the latex within the substance of the SL, as seen in our study with methylene blue on gross anatomy sections, was not described. Bischofberger et al. (3) performed a comparative study of the suspensory ligament origin using magnetic resonance imaging (MRI), ultrasonography, and histology. On sagittal T2-weighted magnetic resonance images, they found high signal intensity assumed to be due to the synovial fluid in the carpometacarpal joint capsule pouches extending 1.41 cm (± 0.25) distally into the SL. This MRI finding was not detailed further or confirmed by comparison to histology. A similar study by Nagy and Dyson (15) using MRI and histology on forelimb suspensory ligaments did not confirm the aforementioned MRI findings but described synovial fluid from the carpometacarpal joint on the axial aspect of the SL and only sometimes between the lateral lobe and fibers attaching to the third metacarpal bone. There was no histological description of synovial tissue within the PSL.

The volume of 10 ml contrast mixture used in this study equals the usual amount of local anesthetics injected into the middle carpal joint for diagnostic purposes (8). Local anesthetics commonly used, such as mepivacaine, have a lower molecular weight and hence potentially a higher diffusion within soft tissues than the contrast medium used in this study, and while this will not affect the distribution within a synovial cavity, it may diffuse more readily within the PSL than the contrast agent. Hence, local anesthetics would be expected to desensitize pathology within the PSL even more efficiently when performing diagnostic local anesthesia of the middle carpal joint.

Contrast enhancement of the SL due to reasons other than a synovial communication, such as diffusion of contrast between tissues or into vessels extending into the ligament, cannot be ruled out but is unlikely to have a significant effect on the marked difference in HU values between the two groups. Following intrasynovial injection, the pool of positive contrast medium was clearly visible on CT images within the SL with sharp borders to the surrounding tissue and was shown to originate from the outpouchings of the CMCJ. In addition, the evidence of synovial tissue within the SL histologically and color pigmentation of the center of the SL lobes on gross anatomy sections allow the conclusion of a true synovial communication, rather than diffusion of contrast medium.

Most of the errors in intra-observer variability in measuring HUs of a structure arise from the inability to accurately delineate the outlines of the PSL. When applying operator-defined ROIs, care must be taken to visualize the suspensory ligament by establishing the outlines repeatedly from proximal to distal using MPR, as well as appropriate window (200–600 HU) and leveling (70–150 HU). Other authors have used this technique to measure mean HUs in tendons and ligaments of the distal limb following contrast medium and found the soft tissue anatomy on CT well-defined (16). In our study, the PSL was clearly demarcated on CT images, could be differentiated from other soft tissues, and allowed visualization of its typical tissue bundle structure, and hence, our intra-observer variability was low (1.97% CV). Horses included in the study had no signs or a known history of pain or pathology associated with the SL, and no abnormalities associated with the PSL were identified on CT. Although only few reports exist in the literature on CT imaging of orthopedic soft tissue injuries, especially affecting the PSL, they have shown CT to be useful in this area. Berner et al. (17) found a good correlation of SL findings obtained with CT compared to MRI in one case of desmopathy of the PSL in the forelimb with a metacarpal cortical fracture in a warmblood gelding. Another case report on suspensory branch desmitis applying CT, MRI, and postmortem examinations among other techniques described that the normal appearance of the PSL and SL body could be recognized on CT (18). In one publication discussing CT imaging for the diagnosis of new bone formation in horses with proximal suspensory desmitis, evaluation of the bone was the main focus as the CT machine used, a peripheral quantitative CT scanner, was not suitable for the assessment of soft tissues in general (19). Magnetic resonance imaging has become widely available and is considered to play a predominant role as a modality for the advanced assessment of soft tissues in the distal limb, such as the proximal suspensory in the forelimb. Depending on circumstances like the lack of availability of MRI, case selection, patient suitability, or the focus of the diagnostic question asked in each individual case, the diagnostic utility of CT for cases with suspected proximal suspensory ligament pathology could be considered. Techniques such as CT arthrography or intra-arterial contrast enhancement can increase the possibilities for CT to aid diagnosis of soft tissue lesions (12, 16). In cases of proximal suspensory desmopathy, all tissue types can show signs of pathology, including muscle, adipose, and collagenous tissues (20). It is therefore possible that the synovial invagination of the SL, highlighted in this study, can also be affected by pathology, which could become apparent with an alteration in size or location of the synovial invagination. This may be useful for the diagnosis of proximal suspensory disease. Further studies evaluating the normal appearance of the proximal suspensory ligament on CT vs. pathology and the histological validation of these findings are necessary to elucidate the clinical utility.

Another clinical implication of the synovial invaginations could be additional information on the etiology of PSL disease. The joint synovium and its inflammation is known to be a predominant feature and cause of degenerative joint diseases, such as rheumatoid arthritis and osteoarthritis (21, 22). The release of pro-inflammatory cytokines from the inflamed synovium *via* invaginations would be detrimental to the PSL. On the other hand, desmitis of the PSL may also have an effect on the synovial fluid of the carpal joint.

The main limitation of this study is that the CT measurements of the two groups were carried out by different CT scanners. HUs characterize the linear attenuation coefficient in tissue relative to water. Therefore, HUs of different tissues are defined to be stable and, to a high degree, independent of the X-ray spectrum (23). However, earlier studies have also shown the HU to be dependent on different CT parameters, such as X-ray kV, CT design, and reconstructing algorithm (24). In this study, while groups 1 and 2 were examined at two different hospital sites using two different CT systems, the same CT design, similar kV settings, and software algorithms were used. Due to different acquisition protocols, the mA settings were significantly different between the two sites. It is possible that HU values were affected by the different settings and physical parameters. The graphs shown in Figure 3 demonstrate a slight difference in baseline HU values between the two groups, which may be due to these differences. However, this will not affect the conclusion of the study as the difference was obvious in *ex vivo* CT arthrography group 1 with 413.7 HU (±30.81) for proximal and 136.34 HU (±4.08) for distal measurements. In the *in vivo* plain CT group 2, the mean of the HU values was only marginally lower proximally at 92.72 HU (±3.03) compared to 106.17 HU (±1.61) for distal, which indicates a higher degree of attenuation of the SL from proximal to distal. The small decrease in the mean HU values of the SL at the levels 1.5–3 cm in plain CT group 2 could be explained by the presence of hypoattenuating muscle and fat tissues seen in the center of the SL, which are more prominent just below the origin than in the SL body (25–27). The amount of muscle tissue in the PSL varies between studies and location (31% in forelimbs and 41% in hindlimbs) (25) but is greater than in the body of the SL (1–11%) (26) and the SL branches (7.5%) (25). The amount of adipose tissue relates to the muscle tissue as it is mainly located between muscle and collagenous parts of the SL (27). Given the anatomical variability of the proximal SL, selecting the contralateral forelimb of the specimens in the CT arthrography group 1 as controls would have reduced possible variations in the measurements between different horses. The results of mean HU measurements of plain CT group 2, as shown in Figure 3, show a comparable distribution of values between the limbs with a homogenous curve progression together with the distal measurements (3–6 cm distal to Mc3) of CT arthrography group 1. The variability of SL mean HU values between animals is therefore expected to be low.

Histological processing removed the water-soluble methylene blue solution, which is to be anticipated. Therefore, the identification of the synovial tissue within the ligament was used as evidence for the presence of the synovial invaginations, rather than being able to determine diffusion. Methylene blue was identified within the suspensory ligament in the gross anatomy section, which was used to target the area for subsequent sampling. Further examination using different staining procedures, which can withstand the histological preparation process (e.g., Indian ink), would allow for drawing a direct link between the synovial invaginations and the contrast distribution within the substance of the PSL.

All limbs were injected with a mixture containing methylene blue. Histology was performed on one limb, and gross anatomy sections were performed on two limbs in this study. Due to practical limitations regarding the preparation processes involved producing histological slides of a ligament close to its insertion onto bone, performing histology on all limbs would have gone beyond the scope of this particular study. Further studies focusing on the histology of the synovial outpouching of the carpometacarpal joint within the SL with examination of a higher number of limbs are needed to elucidate the consistency of the histological appearance of a synovial lined cavity.

In conclusion, we have shown that positive contrast medium is found within the PSL in all horses following injection of the MCJ. These findings indicate a direct synovial communication between the two structures. This was confirmed by identifying a synovial lined cavity histologically within the SL and dye injected together with the contrast medium in gross anatomy sections correlating to the location of contrast within the SL on CT images. The synovial communication provides further clarification of the interaction between perineural and intra-articular diagnostic local anesthesia in the carpal and subcarpal regions.

## Data availability statement

The raw data supporting the conclusions of this article will be made available by the authors, without undue reservation.

## Ethics statement

The animal study was reviewed and approved by Clinical Research and Ethical Review Board at the Royal Veterinary College in London, United Kingdom (URN 2022 2108-3). Written informed consent was obtained from the owners for the participation of their animals in this study.

## Author contributions

CG, RM, and RS contributed to the conception and design of the study. CG organized the database and wrote the manuscript. CG and RM performed the statistical analysis. RT and AF performed and interpreted histology and gross anatomy sections. All authors contributed to the manuscript revision, read and approved the submitted version.

## Conflict of interest

Author CG is employed by Pferdeklinik Hochmoor GmbH. The remaining authors declare that the research was conducted in the absence of any commercial or financial relationships that could be construed as a potential conflict of interest.

## Publisher's note

All claims expressed in this article are solely those of the authors and do not necessarily represent those of their affiliated organizations, or those of the publisher, the editors and the reviewers. Any product that may be evaluated in this article, or claim that may be made by its manufacturer, is not guaranteed or endorsed by the publisher.
